# Description of the CARE4STROKE programme: A caregiver‐mediated exercises intervention with e‐health support for stroke patients

**DOI:** 10.1002/pri.1719

**Published:** 2018-05-24

**Authors:** Judith Vloothuis, Julya de Bruin, Marijn Mulder, Rinske Nijland, Gert Kwakkel, Erwin E. H. van Wegen

**Affiliations:** ^1^ Amsterdam Rehabilitation Research Centre|Reade Amsterdam The Netherlands; ^2^ Department of Rehabilitation Medicine, Amsterdam Movement Sciences VU University Medical Center Amsterdam The Netherlands; ^3^ Amsterdam Neuroscience Vrije Universiteit Amsterdam The Netherlands; ^4^ Department of Physical Therapy and Human Movement Sciences Northwestern University Evanston IL USA

**Keywords:** caregiver, e‐health, exercise therapy, rehabilitation services, stroke

## Abstract

**Background:**

Intensity of practice and task and context specificity are key factors for improving functional outcome in stroke survivors. Novel methods are needed to augment intensity of practice with minimal use of resources and costs. Caregiver‐mediated exercises (CMEs) focused on mobility, in which a caregiver acts as an exercise coach and can increase the intensity of practice. There is preliminary evidence that CME can improve functional outcome, reduce length of stay, and allow early supported discharge, without an increase in caregiver burden. In the CARE4STROKE programme (C4S), CME therapy and e‐health support are combined to promote a smoother transition from the inpatient setting to the home environment, with active rehabilitation continuing in the community.

The objective of this paper is to describe the content of the C4S intervention in detail and explain implementation of this intervention in practice using the Template for Intervention Description and Replication checklist.

**Methods:**

Content, timing and intensity of the programme, participant screening and selection, and intervention procedures were described using the Template for Intervention Description and Replication checklist. Mobility exercises and use of a video application on tablet/smartphone are explained. The role of the caregiver as provider of the intervention is illustrated.

**Discussion:**

C4S prescribes an additional exercise dose of 1,200 min and may be a promising novel and effective method to augment the pallet of therapeutic options for stroke rehabilitation. Important aspects for successful implementation are availability and suitability of a caregiver. Suggestions for additional use of e‐health technology are described.

**Implications for physiotherapy practice:**

The presented description of C4S gives physical therapists practical guidelines to facilitate implementation of the CME intervention.

## 1 INTRODUCTION

Intensity of practice and task and context specificity are key factors of poststroke rehabilitation, because they can improve outcome in terms of mobility and activities of daily living (French et al., 2010; Galvin, Murphy, Cusack, & Stokes, 2008; Kwakkel, 2006; Kwakkel et al., 2004; Langhorne, Bernhardt, & Kwakkel, 2011; Lohse, Lang, & Boyd, 2014; Veerbeek et al., 2014; Veerbeek, Koolstra, Ket, van Wegen, & Kwakkel, 2011) and thereby facilitate early supported discharge (ESD; Fearon & Langhorne, 2012; Langhorne et al., 2011). Caregiver‐ or family‐mediated exercises (CMEs; Galvin, Cusack, O'Grady, Murphy, & Stokes, 2011; van den Berg et al., 2016; Vloothuis et al., 2015; Vloothuis et al., 2016), in which caregivers, such as partners, family members, or friends are actively involved in rehabilitation training, may be a promising and cost‐effective way to augment intensity of daily practice during inpatient stay. CME can continue after discharge to a patient's own home situation and thereby facilitate ESD. A systematic review of nine trials found very low to moderate quality evidence that CME can improve standing balance, walking distance, and quality of life, without increasing caregiver burden, suggesting that CME may augment the pallet of therapeutic options for rehabilitation after stroke (Vloothuis et al., 2016). However, none of these trials included e‐health technology such as telerehabilitation services to support treatment adherence or included exercise apps to support CME. The combination of CME and supported self‐management by using e‐health technology may be a novel way to improve self‐efficacy and empower stroke patients and their families, and reduce caregiver burden (van Vliet, Pomeroy, Wolf, & Kwakkel, 2015).

The CARE4STROKE programme (C4S) combines CME with e‐health support after stroke and is hypothesized to augment intensity of practice, increase functional outcome, and facilitate ESD. One recent Phase II proof‐of‐concept trial tested a similar approach to C4S in Adelaide (Australia; van den Berg et al., 2016). A significant reduced level of caregiver fatigue with increased feelings of self‐efficacy was found at follow‐up. Per protocol analysis showed a reduced length of inpatient stay and fewer readmissions, whereas patients reported a significant improvement of their extended activities of daily living.

Using the same design and the primary and secondary outcomes, an observer‐blinded multicentre randomized controlled CARE4STROKE trial is currently in the analysis stage in Amsterdam to investigate the (cost) effectiveness of CME combined with e‐health tools to improve self‐reported mobility and to reduce length of inpatient stay, caregiver burden, and costs when compared with usual care (Vloothuis et al., 2015).

C4S is a complex rehabilitation intervention (Craig et al., 2008; Langhorne et al., 2011). The description of complex rehabilitation interventions in stroke rehabilitation is typically incomplete (Bernhardt et al., 2016). As a result of the lack of transparency, it is difficult to replicate interventions, properly interpret the effects, and implement promising interventions in clinical practice. The aim of the present paper is, therefore, to describe in detail the essential elements of the C4S intervention using the Template for Intervention Description and Replication (TIDieR) checklist (Hoffmann et al., 2014; Hoffmann et al., 2015; Hoffmann, Erueti, & Glasziou, 2013).

## 2 METHODS

The C4S is an 8‐week exercise intervention, investigated in the multicentre randomized controlled CARE4STROKE trial. A detailed description of trial design, inclusion and exclusion criteria, primary and secondary outcomes, and applied statistics is published elsewhere (Vloothuis et al., 2015). The Medical Ethics Review Committee of the Slotervaart Hospital and Reade approved the study (NL34618.048.12). The trial is registered in the Dutch trial register as NTR4300.

### 2.1 The C4S, description of the intervention according to TIDieR guidelines

Item 1 Brief name of the intervention

CARE4STROKE

Item 2 Why—Rationale of the essential elements of C4S

C4S is a complex rehabilitation intervention, containing several interrelated components (Craig et al., 2008). A comprehensive treatment package is tailored to the individual patient (Langhorne et al., 2011). A detailed description of the rationale behind C4S has been described earlier (Vloothuis et al., 2015).

The main components of C4S are (a) the exercises, which are caregiver mediated and do not replace, but are in addition to usual care, and (b) the use of e‐health tools. The caregiver can, for example, be a partner, family member, or friend of the stroke patient (Vloothuis et al., 2015).

The CMEs are aimed to increase intensity of practice, by being an addition to usual care, and thereby facilitate ESD. They are task specific and specifically focused on general mobility, because independence in transfers and gait is an important component for ESD after stroke (Fearon & Langhorne, 2012; Langhorne et al., 2011; Veerbeek et al., 2014).

**Figure 1:**
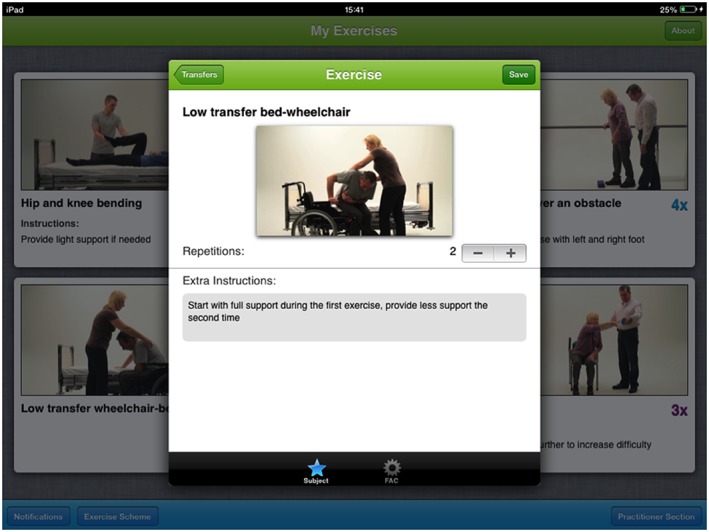
Screenshot of the CARE4STROKE app

The exercises are presented in videos with voice‐over in an e‐health application (“the CARE app”; Figure 1). We hypothesize that this app can support adherence to the programme by patient and caregiver and promote self‐management (Gregory, Alexander, & Satinsky, 2011; White, Janssen, Jordan, & Pollack, 2014).

Safety of both patient and caregiver is a fundamental consideration during CME. New exercises and exercise modifications are practiced with therapist supervision to identify any safety concerns or questions prior to practicing independently. In addition, safety precautions are included in the voice‐over accompanying each exercise video.

Item 3 What—Materials used in the intervention

#### 2.1.1 Exercises

**Figure 2:**
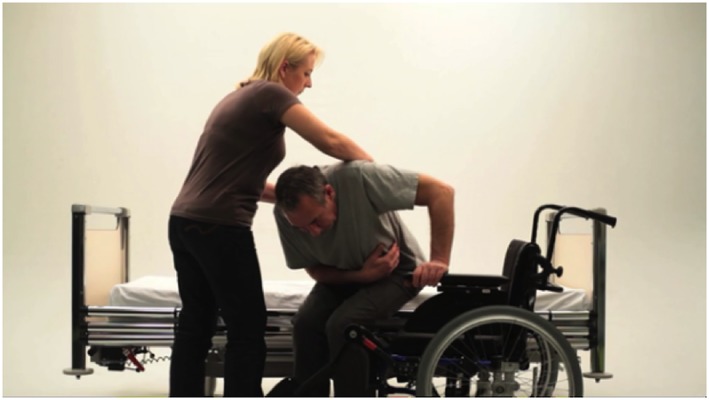
An example of an exercise Transfers: Low transfer from bed to wheelchair. *Aim*: Improve sliding transfer from bed to wheelchair. *Task description for the patient*: The patient sits on the edge of bed. The wheelchair has to stand on the unaffected side. The wheelchair should be at the same level as the bed at a 45° angle to the bed. Armrest and footrest of the wheelchair near the bed should be removed. The break of the wheelchair has to be on. The patient sits up straight with feet supported on ground. The legs are looking away from wheelchair. The feet are placed under knee. The patient leans forward, shoulders directly over knees. The patient reaches and holds with unaffected arm the armrest of the wheelchair. The patient pushes with feet and lift his or her buttock off the bed then slides from bed to the wheelchair. The patient puts the armrest and footrest back into place. *Task description for the caregiver*: If needed, the caregiver places the palms flat on patients back and gives support during movement

Thirty‐seven task‐specific exercises were developed for the purpose of C4S; an overview of these exercises is provided in Table 1 and an example in Figure 2. The exercises, performed 5 times a week for 30 min, are aimed at improving general mobility, including transfers, standing balance, and gait. In addition, there are exercises available for sitting or standing balance, range of motion, and strength exercises for the lower extremity. Subsequently, exercises such as walking outside, stair climbing, walking on uneven ground, and cycling can be trained. The exercises were developed by experienced physical therapists and rehabilitation physicians, with the help of patient–caregiver couples, and proved feasible in a pilot study (unpublished data).

**Table 1:** Exercises categorized by domain and by Functional Ambulation Category (FAC) scale

Domain	Name of exercise	FAC
Lying	Rolling to the affected side	0‐1‐2‐3‐4‐5
	Rolling to the unaffected side	0‐1‐2‐3‐4‐5
	Hip and knee flexion	0‐1‐2‐3‐4‐5
	Ankle towards face and back	0‐1‐2‐3‐4‐5
	Trunk rotation	0‐1‐2‐3‐4‐5
	Bridging	0‐1‐2‐3‐4‐5
	Leg raise	0‐1‐2‐3‐4‐5
	Side line exercise	0‐1‐2‐3‐4‐5
Sitting	Reaching exercise	0‐1‐2‐3‐4‐5
	Look behind you	0‐1‐2‐3‐4‐5
	Buttocks raise	0‐1‐2‐3‐4‐5
	Knee extension	0‐1‐2‐3‐4‐5
	Hip flexion	0‐1‐2‐3‐4‐5
	Practice to stand up	1‐2‐3‐4‐5
Transfers	Transfer from lying to sitting	0‐1‐2‐3‐4‐5
	Transfer from sitting to lying	0‐1‐2‐3‐4‐5
	Low transfer from bed to wheelchair	1‐2‐3‐4‐5
	High transfer from bed to wheelchair	1‐2‐3‐4‐5
	Transfer sit to stand and back	2‐3‐4‐5
	High transfer from bed to wheelchair	2‐3‐4‐5
	High transfer from wheelchair to bed	2‐3‐4‐5
Standing	Standing supported/unsupported	1‐2‐3‐4‐5
	Static balance	2‐3‐4‐5
	Dynamic balance	3‐4‐5
	Squatting	3‐4‐5
	Picking up exercise	3‐4‐5
Walking	Walking with support	1‐2‐3‐4‐5
	Oriented walking	2‐3‐4‐5
	Step exercise	3‐4‐5
	Stair climbing	4‐5
	Different plain walking	4‐5
Other	Cycling on a hometrainer	3‐4‐5
	Cycling on a MOTOmed	0‐1‐2‐3‐4‐5

#### 2.1.2 e‐Health support

The exercises in C4S are presented in an app on a smartphone or tablet computer with touch screen interface allowing independent use by the patient–caregiver couple (Figure 1).

The exercises of C4S are all demonstrated as instructional videos in the app with a voice‐over. The voice instruction leads the patient and caregiver systematically through the exercise.

The app contains a practitioner section and a patient section. In the practitioner section, a tailored exercise programme can be compiled and locked by the therapist. Exercises can be chosen by domain or by Functional Ambulation Category score (Table 1). The number of repetitions can be specified. The order of the exercises can be adapted, and additional instructions can be entered. The therapist can select the affected side of the patient, to match the orientation of video exercises. In the patient section, the selected videos and number of repetitions are displayed, exercise reminders can be set with an alarm, and there is an exercise diary in which the patient can record exercise adherence. In addition, telerehabilitation tools such as videoconferencing and email are used to keep contact between the physical therapist and patient–caregiver couple.

#### 2.1.3 Diary

The patient–caregiver couples are provided with a diary to (a) record daily exercise time, (b) keep notes about the exercises, and (c) record questions for the physical therapist. The format of the diary can be obtained from the authors.

#### 2.1.4 Availability

After the randomized controlled trial is finished, the e‐health application, diary, and guidelines will be made available to the public through an implementation project. Knowledge and experiences to implement the intervention in other settings will be shared. A short introduction film about the exercises can be found at https://youtu.be/pNcmbU9R-A4.

Item 4 What—Procedures

#### 2.1.5 Patient and caregiver selection

Both patient and caregiver should be (a) motivated for CME and (b) able to understand the Dutch or English language. Additional criteria for the patient are as follows: (a) a functional mobility limitation (Functional Ambulation Category < 5), (b) willing and able to appoint a caregiver (with a maximum of two caregivers), and (c) being able to understand and follow instructions. Patients and caregivers with a serious comorbidity that interferes with proper and safe execution of mobility training or with symptoms of depression should not participate.

After informed consent, patients will be asked to appoint one or two preferred caregivers to perform CME with. These caregivers can be asked by the patient, or in consultation with the patient by the treating therapist. It is crucial that both patient and caregiver agree on participation. Thereafter, suitability of the caregiver(s) has to be checked.

#### 2.1.6 Screening session

The screening session is an initial exercise session in which a trained physical therapist evaluates the physical capacities of patient and caregiver, by judging if the couple can perform the exercises safely and adequately and whether the caregiver can physically support the patient. The therapist observes if the patient–caregiver couple can work together and if the caregiver can adequately coach the patient during the exercises. A short checklist, evaluating these criteria, is used by the physical therapist. In case of doubt, the treating physician, the physical therapist in charge, and/or the rehabilitation team can be consulted.

#### 2.1.7 Instruction and evaluation sessions

After enrolment, a 1‐hr session with the patient–caregiver couple and the supervising physical therapist is scheduled to explain the use of the app. In addition, the exercises for the upcoming week are selected by the physical therapist, taking the rehabilitation goals of the patient into consideration, and in consultation with the patient–caregiver couple. The exercises are practiced, the amount of repetitions is set, and the therapist can give additional instructions. The physical therapist hands out the tablet and the exercise diary.

Thereafter, a weekly 30‐min session with the treating physical therapist and the patient–caregiver couple takes place. Exercises of the previous week are evaluated in terms of experienced difficulty and fatigue, and a new or modified exercise programme for the upcoming week can be selected and practiced.

#### 2.1.8 Evaluation of the C4S

After 8 weeks, the effects of participation in the C4S can be measured using validated mobility assessments (Vloothuis et al., 2015).

Item 5 Who—Provider of the intervention

#### 2.1.9 The caregiver

The caregiver acts as an exercise coach by actively supporting and assisting the patient during the task‐specific mobility exercises. This involves both mental and physical support during the exercises. In the sessions with the physical therapist, the caregiver is instructed and trained to give this support. It should be emphasized that the caregiver is not the trainer or therapist.

Caregiver strain is measured before the start of the intervention. During the intervention, the physical therapist closely monitors and discusses caregiver strain. When deemed necessary, based on the professional opinion of the therapist, extra attention is given to the caregiver by the physical therapist or another member of the rehabilitation team.

If desired or more practical, two caregivers can be involved with one participant to divide the time investment of the CME. We set the maximum at two caregivers to ensure optimal practical feasibility and safety, without losing quality of intervention.

#### 2.1.10 The physical therapist

The patient–caregiver couple is supported during the intervention by a physical therapist experienced in treating stroke patients and trained to deliver C4S.

Item 6 How—Modes of delivery

The sessions with the physical therapist and the patient–caregiver couple are individual face‐to‐face sessions. In addition, and specifically after discharge home, the patient–caregiver couple is encouraged to contact the therapist whenever appropriate, using teleconsultation via telephone or videoconferencing and email via the smartphone or tablet computer.

Item 7 Where—Location of the intervention

C4S can be executed in any rehabilitation setting, whether it is in a rehabilitation centre, hospital, nursing home, or the home environment. When patients are discharged during the intervention period, training can continue at home. Most exercises can be executed without any added materials. For some, simple materials such as a ball or chair are needed. In addition, there are exercises in which a staircase, hometrainer, or motor‐assisted stationary bicycle is needed.

Item 8 When and how much

Patient and caregiver are instructed to exercise together, 5 times a week for 30 min preferably including the weekend, during the 8‐week intervention period. With this, a surplus of 150‐min therapy each week, and a total of 1,200 min (8 weeks × 150 min) augmented therapy time, is accomplished. This additional dose is in line with recommendations of evidence‐based guidelines (Veerbeek et al., 2014). In addition, CME allows the patient to train in the weekends as well. Patient and caregiver themselves decide when they exercise, when necessary the physical therapist can help them plan the sessions.

Item 9 Tailoring the programme

During C4S, the physical therapist compiles a tailored exercise programme for the patient–caregiver couple from 37 standardized exercises, choosing those exercises related to the patient goals.

C4S is progressive in nature and is specifically aimed at offering an incremental training regimen (Veerbeek et al., 2014). The physical therapist, therefore, adapts the level of difficulty progressively during the intervention period to be commensurate with the patients' ability. This is achieved by, for example, increasing the number of repetitions or adding instructions for variations.

Item 10 Modifications during the course of the study

The C4S programme is used in the CARE4STROKE trial. No modifications during the course of the trial were made.

Item 11 How well planned—Intervention adherence and fidelity

To measure if participants actually exercised an additional 150 min a week, patients and caregivers fill in a diary. In addition, during the weekly evaluation session, the therapist explicitly inquires about adherence and completing the diary.

For uniform delivery of the intervention, therapists will be trained in a training course with the following content: (a) the inclusion and exclusion criteria, (b) the standardized exercises and the possibilities to customize the CME, (c) therapists role in the screening session, intake exercise session, and weekly exercise sessions, (d) how to fill in the diaries, and (e) the use of the app. In addition, regular retraining sessions will be organized for these participating therapists.

Item 12 How well the intervention was actually delivered

The randomized controlled trial is finished. Patients in the intervention group reported a median of 1,190 min (interquartile range, 870.0–1530.0) of exercise time with a caregiver. This approaches the intended 1,200 min of CME time.

## 3 DISCUSSION

In this paper, we used the TIDieR checklist to systematically describe in detail all key elements of the C4S intervention (Bernhardt et al., 2016; Hoffmann et al., 2013; Hoffmann et al., 2014; Hoffmann et al., 2015). Recently, developing, monitoring, and reporting interventions by using TIDieR were suggested as an important step for improving the quality and transparency of recovery trials after stroke (Walker et al., 2017). The C4S intervention combines CME with e‐health support and aims to augment intensity of daily practice during inpatient stay, continuing after discharge in patient's own living environment, and as such improve functional outcome and facilitate ESD.

A crucial prerequisite for any CME programme is the availability of a suitable caregiver willing to deliver and coach practice. This mutual agreement of patient and caregiver willing to participate is an essential part of C4S and a limiting factor for recruiting potential couples. A strict procedure is described in which the patient appoints the caregiver(s); a caregiver has to meet suitability criteria, *and* a physical therapist gives his accord after the screening session. Only thereafter, the patient–caregiver couple can start with CME. As it is important to know more about the availability of caregivers to participate in CME for future recommendations and implementation of the programme, details about the characteristics of available and suitable caregivers, as well as their perceived strain, will be reported in the CARE4STROKE trial.

C4S could be construed as an extra task for a caregiver in already stressful times (Gordon & Perronne, 2004). However, it has been shown that CME could also decrease caregiver burden and fatigue and increase feelings of self‐efficacy by providing patients and caregivers with more knowledge and education (Galvin et al., 2011; Kalra et al., 2004; van den Berg et al., 2016; Wang et al., 2015). When in doubt about the strain on the caregiver, either before or during the intervention, the treating physician and rehabilitation team should be consulted.

Important aspects to study concerning the availability of a caregiver and the willingness to participate in a CME intervention are cultural, ethnic, and societal differences. For example, the availability of rehabilitation services, travel distances, or the role of the caregiver in society can play an important role. When implementing a CME intervention, these aspects need to be taken into account.

In C4S, CMEs are combined with e‐health technology, by using a mobile application with videos and telerehabilitation services. Despite a lack of trials in this area (Laver et al., 2013), e‐health technology seems promising and is increasingly used (Gregory et al., 2011; White et al., 2014). The functionality and content of the current app and telerehabilitation services in C4S can be expanded. It would be interesting to implement incentives after practice, for example, using text messages or social media to give feedback and a type of reward for patients and caregivers (Harries et al., 2016; Hartin et al., 2016; Jagos et al., 2015). Evaluation and monitoring with built‐in questionnaires or rating scales could be used to monitor difficulty of the exercises, fatigue of the patient, or strain of the caregiver using experience sampling methods (Rickard, Arjmand, Bakker, & Seabrook, 2016). In addition, the paper and pencil diary could be included electronically in the app. This might be more accurate to measure adherence with the programme, especially when combined with wearables.

We are currently analysing the results of the CARE4STROKE trial. Our obtained knowledge about the intervention will be communicated through scientific as well as laymen publications. In addition, a teaching course for health care professionals will be developed in due time in an implementation project.
